# Combination Fillings

**Published:** 1908-06-15

**Authors:** J. J. Travis


### COMBINATION FILLINGS.

BY J. J. TRAVIS, D.D.S.

Read before the Michigan First District Society, March 12th, 1908.

The subject of combination fillings was assigned to me, not because of my peculiar fitness to treat this particular phase of operative dentistry, but, I presume, because it is one of the few subjects not requiring special technical training or manipulative skill, but still of enough general importance to be worthy of consideration by every practicing dentist, regardless of the kind of practice he conducts.

Also because of the fact that in this period of reorganization of the State and local societies, a greater effort seems imperative to assimilate and utilize men from the rural districts.

It was not because of my fitness that I accepted the honor to prepare this paper but rather because I could not with a clear conscience, habitually attend the meetings of the "First District Dental Society" and absorb like a sponge the good things there provided by self-sacrificing fellow practitioners, and refuse, on invitation to contritute my mite in an effort to make our society a living power for professional education and inspiration in our midst.

Therefore, I request that you temper your criticism with charity, not to the extent that you overlook mis-statements and erroneous conclusions allowing me to return to my practice unenlightened, but bear in mind that this is only a suburban contribution.

I am inclined to believe that a paper upon the technique of inserting the comparatively few kinds of reputable combina ion fillings would be uninteresting and uninstructive, but a few facts may be called to mind concerning all restorations involving the combination of two or more materials, whether used as non-conductors or as cement for retention. Consequently I shall include in this discussion not the technique of making and inserting gold and porcelain inlays, but their virtues as preservers of tooth structure, in so far as it is related to other forms of combination fillings depending largely upon cement for retention and sealing.

There are several reasons for employing more than one material for filling the same cavity:

(1) Because by a break in the continuity of the filling or the inter-position of a nonconductor of heat the pulp is protected against thermal changes.

(2) Because different areas of the same cavity often require materials of different hardness or color.

For example, a material that is adapted to filling the gingival portion of a large proximal cavity with friable borders might not have sufficient resistance to sustain the strain of the masticating surface.

(3) By the interposition of adhesive cements we are able not only to save tooth structure by avoiding deep undercuts and retention grooves, but to more perfectly seal a cavity and exclude ever present bacteria and the media on which they thrive.

(4) Because in many instances they are less laborious for the operator and less distressing to the patient.

Any combination which is inserted as so-called permanent work for the express reason that it is easier for the operator or less unpleasant for the patient or consumes less time is usually not wisely chosen and I do not wish that any thing that I may say shall be so interpreted as advocating the use of any "slip-shod," "patch-'em up" methods simply because they are easy.

I wish, however, to refer to some expeditious methods more or less generally employed that are in certain cases as clearly indicated as a porcelain inlay in a labial cavity. For example, at one of our recent meetings there was some discussion as to what was the best filling to preserve the space in deciduous teeth with large proximal cavities.

Some suggested oxyphosphate, some gutta-percha and still others amalgam. The cement was not approved on account of the wasting, and while it preserves the tooth it does not preserve the space. Gutta-percha was not fully approved because it was not hard enough and required too much attention.

Here I think a combination filling is clearly indicated. If you will recall the anatomy of deciduous teeth you may remember that the pulp is comparatively large and the lamina of dentine in the crown between the enamel and pulp very thin consequently the dentinal tubulae are short.

This accounts for the fact that the deciduous teeth do not tolerate large metal fillings as the permanent ones do and some nonconductor is clearly indicated.

If the operator after preparing the cavity will have his assistant mix the amalgam while he mixes the cement, he can first smear a thin layer of cement over the walls and while the cement is still adhesive, insert the amalgam.

In this way there is less necessity for deep undercuts, avoiding much painful drilling, which is a great advantage with children.

We also obtain a nonconductor, more perfect sealing and something that will not wash away and allow the space to be lost.

A combination of cement and amalgam may be used in the coronal and buccal surfaces of molars of low structural development.

In Dr. Kirk's new American System of Operative Dentistry this method is recommended: "Mix a portion of alloy in the usual way but do not express too much mercury. Then mix the cement and when well incorporated add an equal portion of mixed amalgam, mix thoroughly with stiff spatula and introduce as quickly as possible into the cavity under pressure. A portion of the amalgam unmixed with the cement may be used as a veneer and burnished to the borders." The cement gives perfect sealing and adhesion, while the amalgam incorporated retards the washing of the cement.

Where a gold inlay is not possible this I believe to be the best method to preserve the tooth during the "hardening" process.

I have employed this method in only a limited number of cases, but I have been gratified with the results.

I am not contending but that cohesion in both materials is weakened in the mixture, but it seems to combine sufficient strength and adhesion to support teeth of imperfect structure better than either plastic alone. There is probably no operator present who ever uses amalgam but has combined it with cement and well knows the advantages gained.

Many are using cement as a cavity lining and retainer for gold in foil or fiber form, and in this particular form of filling greater opportunity is offered for careless "hurry up" operating than in any other combination with which I am familiar.

Mat or fibre gold is packed into the soft cement with no attempt made for retention other than the adhesive property of the cement. It is not safe to rely upon such a filling with any less mechanical retention than would be necessary for an inlay in the same position.

I am persuaded, however, that if judgment is used in securing mechanical retaining form and not allowing the cement to reach the border, also in allowing sufficient time for the cement to set before malleting a well condensed foil filling can be inserted with less sacrifice of sound tooth structure than the inlay and very closely approaches it in preserving value.

In fact, I think there are places where its superiority may be strongly argued because less cutting away of sound tooth structure is necessary.

Inasmuch as large labial and buccal cavities have been so troublesome to the inlay maker (particularly the matrix inlay and that we nearly always find the enamel around such cavities below the normal hardness, and foil fillings very often reveal recurrent decay about the borders in a distressing ly short time, I have tried a method outlined in the *British Dental Journal* and copied in the *Cosmos* and find it sufficiently valuable to describe here.

"Prepare the cavity as for an inlay except that the walls may be very slightly undercut, then cut small retaining pits on opposite sides.

"Now shape No. 30 foil to the cavity approximately and remove, line the cavity with thin cement and replace the foil as a matrix burnish to place, being careful to force all cement away from the border. Now force pellets or moss fiber into the pits with hand pressure and it is not a disadvantage if the matrix is broken at these points and the cement oozes through under pressure.

"Press moss fiber gold into the cement that oozes through and allow it to set before malleting. The filling may now be finished with moss fiber and No. 30 foil or entirely with pellets."

With a reasonable amount of care no cement can be detected about the borders even with a glass. This filling combines the best features of the foil filling and the inlay, viz: mechanical retention and cement sealing.

My observation has led me to believe more and more firmly each year I practice that some adhesive material interposed between the tooth structure and any of the more permanent filling materials now in use is the most rational method of securing with certainty, at once, both additional support and more perfect sealing of cavities in the teeth.

No other material now in use than cement has any value as a supporter of weak walls. But cement alone dissolves to such an extent that it cannot be exposed to the action of the saliva.

It has been repeatedly demonstrated that no oxyphosphate or oxychloride of zinc or copper is absolutely impervious to water or micro-organisms that may be carried with the water.

Inasmuch as the process of crystallization is never complete when the dam is removed, and the water of crystallization which is incorporated with the glacial phosphoric acid in the liquid in nearly the correct proportions, yet not quite sufficient to complete the crystallization, water is taken up slowly from without to meet this need.

Now, if this non-sterile water in the environment is carried to the interior of the cement mass, bacteria may go with it and consequently must reach the dentinal wall which the cement was designed to protect.

I am aware of the fact that certain manufacturers have made color tests to demonstrate that their cements are impervious, but every practicing dentist who has drilled out old cement fillings knows from the odor that the fact is not proven, also that cultures can be made invariably from protected surfaces of cement that has been long in the mouth.

There are two facts, however; which operate to make this phenomenon unimportant.

First. That these bacteria are inert when removed from their natural habitat,

Second. That by the difficulty of access, they have been reduced to the safety limit.

Under the first fact we may observe that the bacteria associated with caries, seem to be aerobic in nature, and elaborate their solvent acids only in a medium or carbohydrates, but since both air and the carbohydrates are excluded from dentine protected with cement the bacteria are inert.

Under the second fact we may observe that the safety point obtained by the reduction in numbers is not a myth, but a real phenomenon demonstrated in every vital tissue, it is natures resistance depending upon the tone of the tissues locally.

All surgeons recognize this law since perfectly aseptic surgery is impossible and they rely upon nature taking care of a few germs, being careful to reduce the number as low as possible. (These same laws explain the fact that we never find caries in sealed root canals of putrescent or pulpless teeth.)

These two facts of exclusion of air and carbohydrates and reduction of numbers explain why cement has enjoyed the reputation of protecting the dentine against recurrent decay better than any other filling material known to the profession so long as its integrity is preserved.

The problem before us then is either to perfect an insoluble cement or an impervious, tight fitting, "lid to the box" to protect our cement.

Now this "lid" should possess the characteristics of tenacity and hardness, adaptability and color to fill all of the requirements for the varieties of positions in the mouth where we are called upon to make restorations.

Unfortunately no one material possesses all of these characteristics, hence we must choose according to the position and size of the restoration and the probable strain it will be likely to have to sustain.

In the history of the profession many materials and many methods of manipulation of those materials have been tried and abandoned until we now have a method of casting pure gold that promises a greater conquest in operative dentistry than any since the advent of porcelain.

Because of the accuracy of fit and the ductile property of the gold permitting us to burnish over the border, together with its strength and unyielding form establishes it as by far the best restoration for the posterior teeth ever brought to the notice of the profession.

Without a doubt there will be improvements made in the technique and in wax and investment material used, enabling us to make more perfect work, with uniformity, but the principle is right and we can trust the Yankee dentist to perfect it

Porcelain, however, for the anterior teeth, is still queen of the filling materials and nothing as yet has ever approached it. There is, however, one more combination filling no depending upon cement for retention or sealing that is very popular with foil workers, and has proven its right to honorable mention among the well tried combinations—it is tin and gold.

In the approximal cavities of the bicuspids and molars this combination is most useful because of the ease with which tin is adapted to gingival borders.

In these approximal cavities whe e decay has reached nearly to the point of junction of the cementum and enamel, there is liable to be, after the cavity is prepared, a narrow strip of enamel running from lingual to buccal that is very easily chipped away.

A blow of sufficient force to condense gold against this border is very liable to fracture the enamel where its very position makes its detection impossible.

The most carefully inserted filling with this fatal accident at the gingival, will surely fail and no manipulative skill however perfect can *uniformly* avoid this fatal fracture if gold is used and thoroughly condensed.

Soft gold in large pieces is often employed in starting, and the bottom heavily covered before the mallet is used, still we know that there are teeth of low structural development that fracture with a surprisingly light blow, and in as much as tin can be condensed with less force than gold, the danger of fracture is reduced.

Its adaptability is little short of marvelous on account of the flow under pressure, and if an accurately fitted matrix is employed and rigidly retained in position, the foil thus confined may be condensed to sufficient hardness to make an unyielding foundation for a cohesive gold superstructure.

The cavity must be prepared as for an all gold filling, except that the mechanical retentive form must extend right to the occlusal surface, in other words, the gold must have its retaining form independent of the tin.

It is usually customary to fill about two-thirds of the cavity with tin and finish with cohesive gold. If the surface of the tin is flat, that is, if a flat floor is provided for the gold, we avoid any tendency for the gold to slide upon the tin under the pressure of mastication.

Various methods are used in manipulating the tin, some use tin along to build up the first two-thirds of the filling, while others use tin and gold foil rolled together.

I have used both methods quite extensively for the past five years, and personally, I favor rolling the gold and tin together.

I lay down a sheet of No. 4 soft gold foil and upon it a sheet of No. 4 tin foil and then another sheet of gold. I then cut this into halves or thirds and roll together rather tightly. In this manner the gold will be outside, but it does not make any material difference which is outside. There is no foil, either tin or gold, but that is injured by overworking and the less it is torn and worked to pieces in packing the better, therefore, the smoother the surface and the less laceration of the foil the more dense will be the filling.

The idea that one must have a wedging force to drive tin to the borders is, in my opinion, erroneous, for the flow of the metal is an important factor that you do not get with gold and enables one to adapt it more easily.

It is very evident that a wedging force with hand pressure will not bring the molecules as close together as a flat-ended gold plugger under the impact of the mallet. Therefore, while I have not entirely discarded the use of the tin pluggers, I use my gold pluggers far more extensively and think I get a harder filling.

One cannot be too careful in inserting the tin to avoid cutting it up into small pieces, nor is it safe to allow small pieces to be condensed in corners near the matrix as they are liable sometimes to become dislodged, leaving an exposed border.

So I prefer to cut my rolls into strips not over an inch long and starting in one corner fold the foil back and forth until it is all in, condensing well each time it is folded. I usually put in about three layers before condensing and then mallet well after each successive fold.

By this method one gets no small detached pieces at the corners and if carefully placed is, in my judgment, superior to pellets, although I used the pellets exclusively for over four years.

Tin foil oxidizes more readily than gold and its cohesive property is impaired more quickly if left open to the action of the air, moisture and dust in dental offices, yet most operators are very much more careless in keeping it protected from such deteriorating influences.

Cohesive gold may now be used to fill the occlusal third of the molar, making a hard wearing surface, and we have the advantage of filling with gold the easiest portion of the cavity where access is best.

The filling may be finished the same as any approximal gold filling, but the surface of the tin is improved by burnishing.

Some skeptical persons have argued against the use of two metals in the same tooth on the ground of electrical action when the mouth becomes acid, but you will pardon me if I quote the late Dr. Miller on this point. Dr. Miller became familiar as he says with tin alone and in combination with gold, through Dr. Abbott, with whom he was early associated in Berlin and became a strong believer in its virtues. He says: "We may say therefore that neither experimentally, theoretically nor practically can any good or bad results be expected from the electrical action of a tin-gold filling upon the tooth structure, neither have we to fear a disturbance of the pulp."

Since I received notice last summer that I was expected to write upon this subject, I have made many examinations of the tin-gold fillings I have inserted since I began practicing at Plymouth, and I have reason to be gratified over the results that tin has enabled me to obtain, however, I find some fillings very black and a little color showing through the enamel where it is thin.

I have asked a number of operators whom I knew were using tin and their opinion seemed to be that it became dark itself, but did not discolor enamel but slightly, if at all, and was far superior to amalgam in this regard.

Dr. Miller says:

"Tin in the course of a few weeks after insertion undergoes a marked change, the cause of which is not well understood. It results in a discoloration of the filling which sometimes is but slight and at other times completely black; furthermore, in a slight expansion of the filling making it tight even if it was not before.

If we remove the surface of a tin-gold filling, we will find beneath neither tin nor gold, but a semi-crystalline mass which it is sometimes impossible to distinguish from amalgam."

The therepeutic value of tin, if it has such, is probably due to the oxides and sulphides of tin, both of which are antiseptic salts. These salts hermetically seal the cavity ends of the dentinal tubuli either themselves or by the stimulation of secondary deposits of dentine and it is a universally accepted fact that no other metal is so compatible to the dentine as tin.

A note of warning must be sounded to the prophylaxis operator who does not insert tin-gold fillings, that great care must be exercised in the use of the files and scalers upon these fillings, on account of their softness. A pushing motion upon the gingival borders will clean, but do no harm, while a drawing motion will lift the thin portion burnished over the border and shorten the life of the filling.

In conclusion we may observe that although many teeth have been preserved for 30 or 40 years by all cohesive gold foil, yet it seems a mechanical impossibility to be certain every time of obliterating every minute capillary space between the dentine and the foil by mere mechanical skill and concentrated effort.

Since we know that the more minute the capillary crypt he greater is the force of capillarity drawing saliva into it, does it not seem reasonable that our chances of failure are minimized by the more perfect sealing secured by cement?

Therefore, I am inclined to believe that the inlay possessing sufficient strength and adaptability to protect the borders is the highest dental art up to this time, and although I have prided myself greatly upon my foil fillings (for my most concentrated efforts have been with foil), yet I have been forced to recognize in the inlay a strong element of superiority that shall sooner or later establish it as the universally accepted method of filling a very large class of cavities in the teeth.

### DISCUSSION

Dr. Segurd Becker: When I was given this subject I was told that it was on Gold and Tin, but later on I learned it was on Combination Fillings. I find that Dr. Stevens has used for a number of years a method of foil and cement, combining the inlay and filling, and in fact does not use the ordinary foil without that in any cavity, unless it is a little pin-head (filling), and he will use that and porcelain, and his measures I do not think need any comment. In regard to tin and gold. I am afraid tin is going out of use more and more every year; I have found that to be the case in what little I have done on it. I have tried to get a way from tin right along. I have some fillings that I have just removed and put in cast inlays. They were fillings in children and young men and young ladies, at about the age of sixteen, where their parents sent them down to have their teeth filled and I could not see the use of it, and filled them entirely with tin, as their teeth were soft. Upon removing these fillings and putting in gold inlays, I find these teeth comparatively hard, much harder than they were before, and not as sensitive. Certainly tin hardens the inside of the teeth, and I do not believe there is a filling better for the teeth, especially in the early ages, 12 to 16 years. I have had considerable difficulty in getting children to submit to operations on their teeth, so that it has been very difficult to get in cement and amalgam on top of it; I have found, however, that by putting tin in there that I have been able in a short time to get something into that cavity to fill it up. I think Dr. Travis is to be congratulated upon his paper.

Dr. Ward: A great many of the men that I have had occasion to meet, who have criticized the use of tin, have been men who did not know how to use it just as it ought to be. Generally, I find that when tin is used at all it has been supposed that the foil should be heavy, about No. 10, and that it should be inserted with coarsely serrated pluggers, or in order to get it down into the cavity the way it should be, they could best do it with the matrix, and under pressure, and forcibly with the plugger. Prof. Spring, across the water, has done a great deal of work in this connection. He has been able, with a plain surface and hydraulic pressure, to produce alloys equally as good as those produced by heat, with nothing but pure tin and other metals. He can also force tin into the form of a wire as small as No. 40. He has taken alloys of known composition and put them under pressure and forced them through small openings, and the alloys have been identical—showing that the metals in the pure stage, under some pressure, have the property of cohesion. Cohesion of tin and gold are similar. I have not seen a man who is able to do good strong tin fillings that was not pretty much enthused over the tin filling in the mouths of patients, who do not care for their teeth as they should. There is no one thing that will take care of the soft chalky teeth, or that will prevent a recurrent caries in the mouth that is not cared for as it should be, like a tin filling. I think it is used the most successfully in the hands of those who use finely serrated pluggers inserted in the form of No. 4, with a mallet, condensed in the same manner as gold. I think Dr. Hall, of Ann Arbor, can put in a tin filling that has a specific gravity equal to any plug filling that is used. I have seen his fillings that were put in twenty years ago. He always used a finely serrated plugger and goes right after them with a mallet and a well supported matrix.

In regard to the combination of amalgam, gold and cement that the essayist has suggested, there is one thing that has impressed me particularly, the use of cement and amalgam in the deciduous teeth. You all know how hard it is to get retention without making extension. If the buccal surfaces of the upper first molars are used to get an extension, you can get a lining of cement and amalgam, but it is the only place where I have had any success with amalgam and cement combined. I do not believe that the combination is of any great advantage, only in those cases where the retention is doubtful. I think the advent of the development of the gold inlay has unquestionably brought a desirable filling for some cases and for some purposes. I am not enthused over the gold inlay to the extent that some are; while I am having my share of success, I am conservative. I do not think the gold inlay is the thing for everybody nor for every place, nor that everyone that is doing it to-day is the progressive man, while every one that is not doing it is antiquated. We have new things coming along every day, and I think within one or two years we shall just about find out where the gold inlay belongs, the same as we have the porcelain inlay. And many of the men who are still sticking to the gold inlays in every place may lessen the amount of gold inlays and put in more alloys, or lessen the amount of alloys and put in more inlays, but the dentist who is putting in the right plugs where they belong is the man who has the best judgment, and is therefore the best Dentist.

DR. LAND: I want to congratulate the man from the rural district, because I can corroborate the most he has said. I want to tell you that I have inserted amalgam fillings in teeth for forty years. My experience with cement fillings of all kinds has won out in every case. Whether amalgam, tin, gutta-percha, gold or porcelain, is very largely a matter according to age and sensitiveness of the teeth, especially the young teeth. My method for a great many years was to cement red gutta-percha into the temporary teeth, making a pellet and cementing it in, and before the cement hardens, with a warm instrument punch it along, without much pressure to hurt a tender or sensitive patient. I have never been satisfied with any of that class of work. I am now going through a series of experiments in cementing white gutta-percha and I think I will be able to show you before long some very good cases of gutta-percha in keeping the contour. For forty years close observation, I have had more success with gutta-percha than any other known material. I have malleted tin foil, and while I was an enthusiast on tin I used nothing but burnished points. I then looked at the work and was discouraged and then made up my mind that serrations were a humbug. I then went to the other extreme and used a burnished point, and for eighteen years of my life I am using nothing but the burnished point for every kind of filling and contour of every kind. Serrations are an unscientific principle. I went from a crank on gold fillings and became a crank on nearly every kind of a filling. If I was to fill a temporary tooth to-day, I should use gutta-percha, the red gutta-percha with red sulphate of mercury in it, and that will preserve the teeth better than any other substance I know of. The red will stand while the white disintegrates readily, as the oxide of zinc has a bad effect on the gutta-percha. I intend to investigate the best things to mix with gutta-percha in order to make good contour, and this is a thing I am working on now. I will just make one or two remarks in regard to the reason why fillings with cement are better than fillings made without it. The oxyphosphate cement especially does add to the normal tooth structures strength, that is positively proven, and any one who says that it does not, don't know how to use it. The oxyphosphate can be separated. There is no doubt but if you protect it like we do on all our crown and inlay work, you can remove those seventeen or eighteen years afterwards, and find your cement adhering to normal tooth structures, and yet a large number of our failures are due to the fact that we have set the cement to abnormal tooth structures, and as phosphoric acid has such an affinity for organic matters it combines with it and the filling becomes disengaged. A close joint made with an indestructable filling cannot wash out of the tooth except superficially. If it did, you would not have a bridge or inlay of any description worth putting in. Even an acid will not decompose it because it is too small a surface. The first acid action on the narrow surface would destroy that portion and then the acid would become neutral.

Dr Graham: I want to compliment the essayist of the evening on his paper. He said one time that he never saw recurring decay under a tin filling. I believe the majority of us can say nearly as much. About a month ago I saw a filling, a composition of gold and some other metal; the gold was most beautifully contoured, and I had occasion to inspect it because it was in a very bad condition from pyorrhoea. I did not use instruments, simply shoved it ous with my finger. In that tooth there was a combination of gold and some other metal—I thought it was a combination of gold and tin. The gold was beautifully inserted and contoured, and I though I would have a little joke on the essayist to-night in bringing that tooth here. I showed it to my friend Giffen, and he informed me that it was amalgam, instead of tin, and on closer examination I found that to be true. I think we will all agree with the essayist that the recurrence of decay is seldom seen underneath the tin filling, and although from appearances we might expect a recurrence of the decay, we will find upon drilling into that tooth that it is not decay, but a discoloration, and on more than one occasion I have done that thing, and on drilling into the tooth suspecting that there was decay underneath I only found that it was a tin filling. About the combination of cement with all fillings, and particularly with gold. For some time I have used a cement lining under all fillings, and have had my share of failures. When I was in college Dr. Young told us that the proper thing to do was to insert cement under all fillings. Whether I got the wrong conception of his technique or not, I returned to my office and attempted to do that thing. My trouble was in inserting too much cement. If I had a large filling I would insert possibly one-third of the filling with cement, apply my gold or amalgam before it was very hard. I would have very disagreeable results. Since then I have learned to do differently. Now I have better results by applying a very fine smear of cement, not more than the quantity that you would apply with a well fitting inlay. If your cavity is so large that you want to take up a good deal of the space with cement, I prefer to make two mixes of cement. Apply quick setting cement first and fill to the required fullness, allow this to harden, which will not take long, and then before inserting the gold apply another thin layer of cement. In that case you protect your margins, and I at least get better results. That has been my lesson.

Dr. Bunting: I want to congratulate Dr. Travis on what he said in his excellent paper. I feel that it has been worth my while, and all of us, to come to hear this paper. I wish to make a plea for myself. I have been doing a little work along this line. I have a machine for slicing tooth sections for microscopial work, and have been working lately on the subject of filled teeth. We get at the college teeth of all kinds, and I have sections of about 125 or 130 teeth and from these sections I see some surprising things about which I am entirely at sea at the present time. I have not enough material to complete the work and put it in such a shape that we can come to any definite conclusions, but the thing that troubles me most is the lack of material. I wish I had more filled teeth. In these teeth sent to us there are very few gold fillings. When a tooth is extracted with any sized gold filling, the filling is lost or gone. I would be glad if the men here to-night would save me filled teeth of all kinds, I should be glad to pay for any gold that is in the teeth sent; the teeth are worth more to me than the gold they contain. I want something besides amalgam fillings, which is the most bulk of what I have. There have been only a very small percent of amalgam fillings in which I am absolutely sure that the decay came in from the outside. It looks in a large percentage of the cases as if the decay was left in the bottom of the cavity and started in the base of the tooth, the whole filling being apparently tight around the border. Dr. Travis said decay will not occur where there is an absence of hydrocarbons or air. I cannot contest that, but this I know; the teeth of sheep have been drilled and carious dentine has been packed, a small amount in the bottom of the cavity and it has been filled with a tight filling. These cavities have been opened again in a few months time when it was found that a large cavity has been formed underneath that filling—a tight filling and carious dentine developed underneath.

Dr Travis: I am glad that Dr. Bunting brought out the point in regard to recurrent decay. We most of us believe that this recurrent decay has been due to the slovenly preparation of the cavity. I thought when he started that he was going to tell us of some slides that he has made of sections of tin fillings. I know that in talking with Dr. Hoff about this matter, he told me that he noticed a great difference in the texture of the enamel and the dentine under amalgam fillings and under tin fillings that have been in place for a number of years; under the amalgam fillings it was very much checked, and in the tin fillings there seemed to be an obliteration of the dental tubuli but there does not seem to be that checked condition. I do not remember to have ever seen recurrent decay under tin fillings; perhaps this is due to the fact that I have not made sufficient observations in this line. Most of the tin fillings that I have observed have been fillings put in by myself within five years, so that there may be lots of it in five years more. None of those operating near by me are using tin, so I see very little of it that I do not insert myself. It has not had a long enough trial as yet to determine whether this is true.

Another point I want to call to mind, is that the profession should be educated a little bit on this line. There are men who are not using tin who will see a filling that some other operator has put in with tin, and when they see that dark color underneath, they immediately proceed to take out the filling and then find that it is all right. That is a bad feature, and it seems that the dentists ought to take time enough to examine the fillings to see what they are working with, and not take out a filling that is perfectly sealed in the tooth and all right.

Dr. Ward brought out the point that tin and gold, any of these pure metals can be welded with alloys, simply by pressure. Dr. C. N. Johnson in his work on that subject says any desired alloy made of a composition of gold and tin, when worked together and well packed, does not form an alloy at once, but it takes the form of an alloy when it has been in the tooth for some time. When such fillings are taken out they are found to be neither gold nor tin, but an alloy. The alloy is formed because of the conjunction of the two metals.

